# Cumulative scores based on plasma D-dimer and serum albumin levels predict survival in esophageal squamous cell carcinoma patients treated with transthoracic esophagectomy

**DOI:** 10.1186/s40880-015-0062-2

**Published:** 2016-01-11

**Authors:** De-Qing Liu, Fang-Fang Li, Wei-Hua Jia

**Affiliations:** State Key Laboratory of Oncology in South China, Collaborative Innovation Center for Cancer Medicine, Sun Yat-sen University Cancer Center, 651 Dongfeng East Road, Guangdong, 510060 Guangzhou P.R. China; Department of Radiation Oncology, Chongqing Cancer Institute, Chongqing, 400030 P.R. China

**Keywords:** DA score, Esophageal squamous cell carcinoma, Survival, Prognostic factors, Transthoracic esophagectomy

## Abstract

**Background:**

Recently, studies have shown that plasma D-dimer and serum albumin are prognostic markers for esophageal cancer. The purpose of this study was to evaluate a novel prognostic scoring system—DA score (combination of preoperative plasma D-dimer and serum albumin levels)—and analyze the association between survival of patients with esophageal squamous cell carcinoma (ESCC) and their Glasgow prognostic score.

**Methods:**

In this retrospective study, preoperative biochemical markers and clinicopathologic factors in 260 ESCC patients treated with transthoracic esophagectomy were reviewed. According to receiver operating characteristic analysis, the cutoff values of D-dimer and albumin were defined as 0.5 μg/mL and 43.8 g/L, respectively. Patients with high D-dimer levels (≥0.5 μg/mL) and low albumin levels (<43.8 g/L) were assigned a score of 2, those with only one of the two abnormalities were assigned a score of 1, and those with neither of the two abnormalities were assigned a score of 0.

**Results:**

ESCC patients with a DA score of 0, 1, and 2 numbered 55, 116, and 89, respectively. Survival analysis showed that patients with a DA score of 2 had lower overall survival (OS) rates than those with DA scores of 1 and 0 (37.1% vs. 52.6% and 76.4%, *P* < 0.001); similar findings were observed for disease-free survival (DFS) rates (32.6% vs. 44.8% and 67.3%, *P* < 0.001). In addition, the predictive value of the DA score was also significant in patients with stages I–IIA and stages IIB–IV ESCC. Multivariate Cox regression analyses indicated that hazard ratios (HRs) for predicting OS of patients with DA scores 1 and 2 were 2.25 (*P* = 0.010) and 3.14 (*P* < 0.001), respectively, compared with those with a DA score of 0, and HRs for predicting DFS of patients with DA scores of 1 and 2 were 1.86 (*P* = 0.023) and 2.68 (*P* < 0.001), respectively, compared with those with a DA scores of 0.

**Conclusions:**

Our study suggests that preoperative DA scores are notably associated with postoperative survival of ESCC patients.

## Background

Esophageal cancer (EC) is the eighth most common cancer worldwide and the sixth leading cause of death due to cancer [1]. In China, the predominant histological type of EC is esophageal squamous cell carcinoma (ESCC), which accounts for 90% of ECs [2–4]. Despite multidisciplinary treatments that have been developed for ESCC, including surgical resection and neoadjuvant chemoradiation therapy, transthoracic esophagectomy remains the primary therapeutic strategy for locoregional ESCC [5–7]. Unfortunately, even after surgical resection, ESCC patients still have a high postoperative recurrence rate and poor prognosis, with only a 5-year survival rate ranging 26.2%–49.4% [8]. Therefore, identifying a simple and instructive indicator that could identify ESCC patients with high risk of tumor recurrence and poor prognosis is urgently needed.

To date, clinical stages, pathologic findings, and performance statuses have been considered the predominant prognostic factors in patients with common solid tumors. However, clinical staging is often inaccurate despite the use of high-resolution computed tomography (CT), and definition of performance status is sometimes subjective [9, 10]. Recently, the Glasgow prognostic score (GPS), which is based on C-reactive protein (CRP) and albumin levels as well as reflects inflammation and nutritional status, was used as a prognostic indictor for numerous cancers [11]. However, some studies indicated that systemic inflammation and malnutrition are not significant in operable ESCC patients, leading to decreased clinical application of GPS [12]. Therefore, it is imperative to identify a novel prognostic scoring system that can improve the clinical outcome of ESCC patients.

In previous studies, coagulation abnormalities were commonly seen in patients with malignant tumors [13–15]. Systemic activation of blood coagulation and procoagulant changes were associated with angiogenesis, tumor cell invasion and progression, and metastatic spreading [16]. D-dimer, which is an end-degradation product of fibrin, indicates the activation of hemostasis and the hypercoagulable state. Elevated D-dimer levels were observed in acute venous thromboembolism, pregnancy, infectious diseases, surgery, as well as cancers [17]. Moreover, recent studies showed that elevated D-dimer levels predicted poor prognosis in various types of cancer, including breast cancer, lung cancer, ovarian cancer, and EC [16, 18]. Therefore, we hypothesized that the combination of D-dimer and albumin, which reflects malnutrition and poor survival in cancer patients [19–21], may provide a simple and objective prognostic scoring system for ESCC patients. In this study, we devised a novel prognostic scoring system, called the DA score (combination of D-dimer and albumin levels), and analyzed the clinical significance of the DA score for predicting the prognosis of ESCC patients who underwent transthoracic esophagectomy.

## Methods

### Patient selection

In this study, the data of 260 ESCC patients who underwent transthoracic esophagectomy between December 2004 and December 2010 in Sun Yat-sen University Cancer Center, Guangzhou, China were analyzed retrospectively. The criteria for case selection were as follows: (1) pathologic diagnosis of primary squamous cell carcinoma; (2) no history of preoperative adjuvant therapy; and (3) preoperative plasma D-dimer levels, serum albumin levels, and serum CRP levels obtained within 1 week before surgery. The following data were also recorded for all patients: age, sex, tumor diameter, tumor location, TNM stage, T category, N category, pathologic grade, history of hepatitis B virus infections, and family history of cancer. TNM classification was evaluated using the 6th edition of the Union for International Cancer Control staging. This study was approved by the medical ethics committee of Sun Yat-sen University Cancer Center, and informed consent was obtained from all patients.

### D-dimer, albumin, and CRP measurement

As a part of clinical routine examinations, pretreatment plasma D-dimer levels, serum albumin levels, and CRP levels were measured 24 h to 1 week before surgery. Plasma D-dimer values were analyzed by a latex-enhanced immunoturbidimetric assay and a Sysmex CA 7000 system (Sysmex Corporation, Japan) according to the manufacturer’s instructions. Serum albumin levels and CRP levels were measured by using a Hitachi 7600-020 automatic biochemical analyzer (Hitachi, Japan). The optimal cutoff values of D-dimer and albumin in our study were verified using Youden’s index (YI) from the receiver operating characteristic (ROC) curve for ESCC prediction. The survival status was inserted into the YI to define the cutoff value as described by Huang et al. [22] and Liu et al. [23]. A high level of preoperative plasma D-dimer was defined as ≥0.5 μg/mL, and a low level of preoperative serum albumin was defined as <43.8 g/L.

### D-dimer and albumin (DA) score

ESCC patients with high D-dimer levels (≥0.5 μg/mL) and low albumin levels (<43.8 g/L) were assigned a score of 2, those with only one of the two biochemical abnormalities were assigned a score of 1, and those with neither of the two biochemical abnormalities were assigned a score of 0.

### GPS

GPS was defined as described previously [11]. Specifically, patients with low CRP (<10 mg/L) and high albumin levels (≥35 g/L) were assigned a score of 0, those with one of the two biochemical abnormalities were assigned a score of 1, and those with both high CRP levels and low albumin levels were assigned a score of 2.

### Follow-up

Postoperative follow-up was performed by using gastroscopy and CT scanning every 3–6 months for the first 3 years, then annually until death or the end of the present study. In total, 260 ESCC patients were followed up for 2–91 months, with a median follow-up time of 40.5 months and a median survival time of 35 months (1–91 months). The last scheduled follow-up was on April 20, 2015. Overall survival (OS) was defined as the interval from the date of surgery to the date of death from any causes. Disease-free survival (DFS) was defined as the interval from the time of surgery to the date of local relapse or distant metastasis.

### Statistical analysis

Statistical analysis was performed using the SPSS 16.0 software package (SPSS, Chicago, IL, USA). The Chi square test was used to assess the association between the DA scores and clinicopathologic variables of ESCC patients. OS and DFS curves were plotted by using the Kaplan–Meier method and evaluated by using the log-rank test. Univariate and multivariate survival analyses were performed by using the Cox regression model. Differences were considered statistically significant when the *P* value was <0.05.

## Results

### Patient characteristics

The characteristics of the 260 ESCC patients are shown in Table 1. The median age was 59 years (range, 39–83 years), and 83.5% of patients were males. Tumors were observed in the upper-thoracic esophagus in 8.1% of patients, in the mid-thoracic esophagus in 63.5% of patients, and in the lower-thoracic esophagus in 28.5% of patients. The median diameter of the tumor was 4 cm (range 1–10 cm). The histopathologic grades were well/moderate in 68.8% of patients and poor in 31.2% of patients. Of the 260 patients, tumor invasion depths of T1/T2 and T3/T4 were observed in 50 (19.2%) and 210 (80.8%) patients, respectively; 153 (58.8%) had positive lymph node metastases; and only 3 (1.2%) had distant metastases (M1) before surgery, and metastasis sites were completely excised. Before treatment, 158 (60.8%) patients were diagnosed with stage IIB disease or higher. Hepatitis B surface antigen (HBsAg) was positive in 19 (7.4%) of 257 patients; three patients were absent of the status of HBsAg. Of the 260 patients, 24 (9.2%) reported a first-degree family history of EC.

**Table 1:** Preoperative DA score and clinicopathologic variables in 260 patients with ESCC

Characteristic	No. of patients	Score 0 (*n* = 55)	Score 1 (*n* = 116)	Score 2 (*n* = 89)	*P* value
Sex					0.414
Female	43	6 (14.0)	22 (51.2)	15 (34.9)	
Male	217	49 (22.6)	94 (43.3)	74 (34.1)	
Age (years)					<0.001
≤59	138	43 (31.2)	61 (44.2)	34 (24.6)	
>59	122	12 (9.8)	55 (45.1)	55 (45.1)	
Tumor location					0.387
Upper-thoracic esophagus	21	4 (19.0)	10 (47.6)	7 (33.3)	
Middle-thoracic esophagus	165	36 (21.8)	79 (47.9)	50 (30.3)	
Lower-thoracic esophagus	74	15 (20.3)	27 (36.5)	33 (43.2)	
Tumor size (cm)					0.079
<4	126	27 (21.4)	64 (50.8)	35 (27.8)	
≥4	134	28 (20.9)	52 (38.8)	54 (40.3)	
Pathologic grade					0.383
I/II	179	36 (20.1)	85 (47.5)	58 (32.4)	
III	81	19 (23.5)	31 (38.3)	31 (38.3)	
TNM stage					0.390
I–IIA	102	22 (21.6)	50 (49.0)	30 (29.4)	
IIB–IV	158	33 (20.9)	66 (41.8)	59 (37.3)	
T category					0.373
1 + 2	50	13 (26.0)	18 (36.0)	19 (38.0)	
3 + 4	210	42 (20.0)	98 (46.7)	70 (33.3)	
N category					0.695
0	107	22 (20.6)	51 (47.7)	34 (31.8)	
1	153	33 (21.6)	65 (42.5)	55 (35.9)	
HBsAg					0.052
Negative	238	46 (19.3)	109 (45.8)	83 (34.9)	
Positive	19	8 (42.1)	5 (26.3)	6 (31.6)	
Family history^a^					0.032
No	236	45 (19.1)	109 (46.2)	82 (34.7)	
Yes	24	10 (41.7)	7 (29.2)	7 (29.2)	

All values are presented as numbers of patients followed by percentages in parentheses

*DA score* D-dimer and albumin score, *ESCC* esophageal squamous cell carcinoma

^a^Patients whose first-grade relatives have a history of esophageal cancer

### Association between preoperative DA scores and clinicopathologic characteristics of ESCC

In the total study population, the median preoperative plasma D-dimer level was 0.5 (25th–75th percentile: 0.3–0.7) μg/mL, and the median preoperative serum albumin level was 43.1 (25th–75th percentile: 40.7–45.0) g/L. Of the 260 patients, 138 (53.1%) showed elevated preoperative D-dimer levels (≥0.5 μg/mL), and 156 (60.0%) showed decreased preoperative albumin levels (<43.8 g/L). Also of the 260 patients, 55 (21.2%) had a preoperative DA score of 0, 116 (44.6%) had a preoperative DA score of 1, and 89 (34.2%) had a preoperative DA score of 2 (Table 1). The association between preoperative DA scores and postoperative clinicopathologic characteristics is shown in Table 1. Older patients had higher preoperative DA scores than younger patients (*P* < 0.001), whereas patients with a family history of EC had lower preoperative DA scores (*P* = 0.032).

### Association between preoperative DA scores and the survival of ESCC patients

**Fig. 1:**
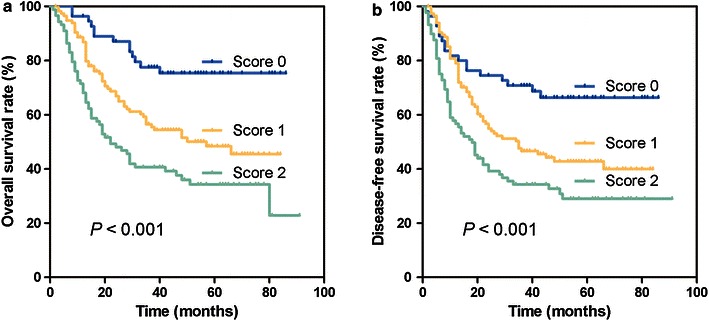
Preoperative D-dimer and albumin score (DA score) and postoperative survival of 260 patients with esophageal squamous cell carcinoma (ESCC). Patients with higher DA scores had significantly shorter OS (**a**, *P* < 0.001) and DFS (**b**, *P* < 0.001) than patients with lower DA scores

**Fig. 2:**
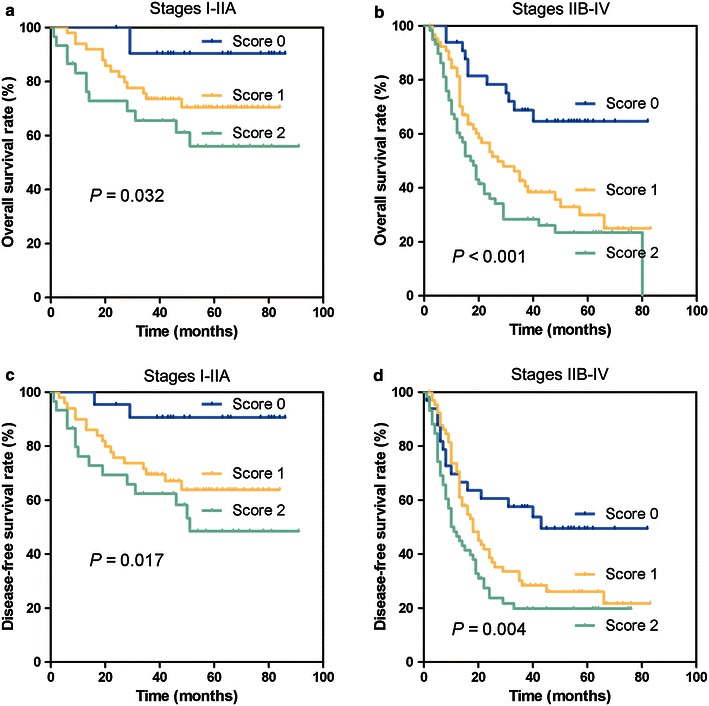
Survival analyses of DA scores in patients with stages I–IIA and stages IIB–IV ESCC. OS curves of patients with **a** stages I–IIA and **b** stages IIB–IV diseases and DFS curves of patients with **c** stages I–IIA and **d** stages IIB–IV diseases are shown according to distinct DA score

In total, 260 patients with ESCC were followed up for 91 months; they had a median survival time of 35 months (1–91 months). Patients with higher preoperative DA scores had a shorter OS than those with lower DA scores (37.1% for patients with a DA score of 2 vs. 52.6% and 76.4% for those with DA scores of 1 and 0, respectively, *P* < 0.001; Fig. 1a); similarly, DFS was significantly shorter in patients with higher DA scores than in those with lower DA scores (32.6% for patients with a DA score of 2 vs. 44.8% and 67.3% for those with DA scores of 1 and 0, respectively, *P* < 0.001; Fig. 1b). To assess the confounding effect of ESCC on the clinical stage, we further stratified patients into different groups based on TNM stage (stages I–IIA and stages IIB–IV). The results showed that the DA score in patients with stages I–IIA and IIB–IV tumors were independently associated with OS (stages I–IIA, *P* = 0.032; stages IIB–IV, *P* < 0.001; Fig. 2a, b) and DFS (stages I–IIA, *P* = 0.017; stages IIB–IV, *P* = 0.004; Fig. 2c, d).

Univariate analysis using the Cox proportional hazard model showed that age, TNM stage, T category, N category, GPS, and DA scores were significantly associated with OS; age, TNM stage, N category, and DA scores were significantly associated with DFS (Table 2). Although preoperative GPS was significantly predictive of OS (*P* = 0.033), high CRP levels (>10 mg/L) and hypoalbuminemia (<35 g/L) were observed in only 39 (15.0%) and 2 (0.8%) of 260 ESCC patients, respectively. Besides, only one patient was assigned a score of 2.

**Table 2:** Univariate Cox regression analysis for survival of ESCC patients

Variable	Overall survival		Disease-free survival	
	HR (95% CI)	*P* value	HR (95% CI)	*P* value
Sex		0.846		0.576
Females	1		1	
Males	0.96 (0.60–1.52)		1.14 (0.72–1.79)	
Age (years)		<0.001		<0.001
≤59	1		1	
>59	2.48 (1.73–3.57)		1.81 (1.30–2.52)	
Tumor location		0.338		0.141
Upper-thoracic esophagus	1		1	
Middle-thoracic esophagus	1.51 (0.73–3.13)		1.72 (0.87–3.41)	0.119
Lower-thoracic esophagus	1.20 (0.55–2.60)		1.30 (0.63–2.70)	0.479
Tumor size (cm)		0.291		0.276
<4	1		1	
≥4	1.21 (0.85–1.72)		1.20 (0.86–1.67)	
Pathologic grade		0.423		0.534
I/II	1		1	
III	1.17 (0.80–1.69)		1.12 (0.78–1.59)	
TNM stage		<0.001		<0.001
I–IIA	1		1	
IIB–IV	3.16 (2.07–4.83)		3.24 (2.19–4.80)	
T category		0.018		0.051
1 + 2	1		1	
3 + 4	1.89 (1.12–3.21)		1.59 (0.99–2.52)	
N category		<0.001		<0.001
0	1		1	
1	2.97 (1.97–4.47)		3.12 (2.13–4.57)	
HBsAg		0.199		0.802
Negative	1		1	
Positive	0.584 (0.26–1.33)		1.08 (0.58–2.00)	
Family history^a^		0.486		0.271
No	1		1	
Yes	0.79 (0.40–1.55)		0.70 (0.37–1.33)	
Glasgow prognostic score		0.033		0.169
0	1		1	
1	0.99 (0.60–1.62)	0.951	0.89 (0.56–1.43)	0.633
2	14.70 (1.95–110.58)	0.008	6.26 (0.86–45.63)	0.070
DA score		<0.001		<0.001
0	1		1	
1	2.50 (1.37–4.59)	0.003	1.94 (1.15–3.27)	0.013
2	4.12 (2.25–7.54)	<0.001	2.99 (1.77–5.08)	<0.001

*HR* hazard ratio, *CI* confidence interval; other abbreviations as in Table1

^a^Patients whose first-grade relatives have a history of esophageal cancer

Furthermore, multivariate Cox regression analysis indicated that DA score, age, and clinical stage were significantly associated with OS (*P* = 0.002, *P* = 0.001, *P* < 0.001, respectively) and DFS (*P* = 0.002, *P* = 0.030, *P* < 0.001, respectively) in ESCC patients (Table 3). Compared with the patients with a DA score of 0, hazard ratios [HRs, 95% confidence intervals (CIs)] for predicting OS of patients with DA scores of 1 and 2 were 2.25 (1.21–4.17, *P* = 0.010) and 3.14 (1.66–5.92, *P* < 0.001), respectively, and HRs (95% CIs) for predicting DFS of patients with DA scores of 1 and 2 were 1.86 (1.09–3.17, *P* = 0.023) and 2.68 (1.54–4.66, *P* < 0.001), respectively, compared with those with a DA score of 0.

**Table 3:** Multivariate Cox regression analysis for survival in ESCC patients

Variable	Overall survival		Disease-free survival	
	HR (95% CI)	*P* value	HR (95% CI)	*P* value
Age (years)		0.001		0.030
≤59	1		1	
>59	1.95 (1.34–2.85)		1.46 (1.04–2.06)	
TNM stage		<0.001		<0.001
I–IIA	1		1	
IIB–IV	3.03 (1.97–4.66)		3.12 (2.10–4.64)	
Glasgow prognostic score		0.217		0.466
0	1		1	
1	0.96 (0.57–1.61)	0.880	0.84 (0.52–1.37)	0.492
2	6.00 (0.79–45.72)	0.084	2.75 (0.37–20.31)	0.322
DA score		0.002		0.002
0	1		1	
1	2.25 (1.21–4.17)	0.010	1.86 (1.09–3.17)	0.023
2	3.14 (1.66–5.92)	<0.001	2.68 (1.54–4.66)	<0.001

Abbreviations as in Tables 1 and 2

## Discussion

In the present study, we developed a novel prognostic scoring system, DA score, based on plasma D-dimer and albumin and assessed its prognostic values in ESCC patients treated with transthoracic esophagectomy. The presented data demonstrate that the preoperative DA score was significantly associated with postoperative survival and could act as an independent prognostic marker for ESCC patients. Furthermore, after stratification by TNM stage, the preoperative DA score remained of great prognostic value in patients with early-stage (stages I–IIA) and advanced-stage (stages IIB–IV) diseases. Therefore, according to the preoperative DA score, which was independent of TNM stage, we can preemptively identify patients who are at increased risk of recurrence and who would have poor prognosis postoperatively; thus, additional treatment, such as neoadjuvant chemotherapy and close follow-up, could be recommended.

GPS, an inflammation-based prognostic score, is widely recognized as a prognostic indicator for a variety of cancers, including liver, lung, breast, colorectal, and gastric cancers [24–27]. However, the clinical utility of GPS in EC patients remains controversial. Furthermore, some studies reported that inflammation and CRP levels are not commonly increased in ESCC patients [12]. Therefore, we evaluated the use of plasma D-dimer levels as a replacement. D-dimer, a degradation product of fibrin, is an indicator of hypercoagulability and endogenous fibrinolysis. Recently, studies revealed that D-dimer associated with cellular signaling systems and could promote cell growth and induce angiogenesis [28]. Buller et al. [29] reported that D-dimer could also induce the spread of tumors by stimulating tumor cells to adhere to endothelial cells. Moreover, Diao et al. [18] recently demonstrated that elevated plasma D-dimer level was associated with the progression of tumor and poor prognosis in EC, which was consistent with previous studies of colorectal, gastric, breast, and lung cancers [16, 23]. In addition, it is well accepted that malnutrition is a predictor of survival in cancer, and serum albumin is considered a nutritional indicator [20, 21]. Previous studies showed that hypoalbuminemia was associated with poor survival in numerous solid cancers, including gastrointestinal, lung, and breast cancers [19, 30]. Therefore, cumulative scores based on preoperative D-dimer and albumin levels may be a potentially predictive marker for prognosis of ESCC patients.

In recent years, some studies demonstrated that GPS was significantly associated with OS and DFS and could act as an independent prognostic marker for EC patients [31, 32]. In our study, preoperative GPS was also significantly predictive of OS in patients with ESCC (*P* = 0.037); however, the present data showed that only 1 patient had a GPS of 2, resulting in decreased benefit for predicting prognosis. As mentioned above, the severity of systemic inflammation and malnutrition in resectable EC might be insufficient to be effective as prognostic markers. Furthermore, multivariate analysis showed that GPS was not independently associated with OS or DFS. Matsuda et al. [12] reported similar results, of which only 2 of 199 EC patients were assigned a GPS score of 2, and GPS was shown to not be an independent prognostic factor for OS or DFS. The discordance in these studies might be explained by three factors. First, the difference of selected objects such as resectable and unresectable EC might lead to various results. Second, ethnicity and epidemiological differences may be the cause of the discrepancies between previous reports and our study. Third, whether the patients accepted neoadjuvant treatment may explain the observed discordance. In this study, the D-dimer and albumin optimal cutoffs were defined as 0.5 μg/mL and 43.8 g/L, respectively, according to ROC analysis, and preoperative D-dimer and albumin levels were both significantly associated with OS and DFS in ESCC patients (data not shown). Thus, we devised a novel prognostic scoring system called DA score and found that the preoperative DA score was significantly associated with age and family history of EC, whereas there was no association between the DA score and TNM stage, T category, or N category, indicating that the preoperative DA score reflected the patient’s individual status rather than tumor burden and progression. Kaplan–Meier analysis showed that patients with high preoperative DA scores had shorter OS and DFS compared with those with low preoperative DA scores. Multivariate analysis demonstrated that DA scores, along with age and TNM stage, were independent prognostic factors for postoperative survival. Therefore, preoperative DA score was superior to GPS as a predictive factor in ESCC patients treated with transthoracic esophagectomy.

We acknowledge that our study has the following limitations. First, our study was a retrospective study with a small sample size. Second, the present data were from a single institution. In addition, we did not include patients who underwent neoadjuvant treatment before surgery, which may have influenced our results.

## Conclusions

In conclusion, our findings demonstrate that the preoperative DA score is associated with survival and can serve as an independent prognostic indicator in ESCC patients treated with transthoracic esophagectomy. We also conclude that DA score is superior to GPS as a predictive factor in patients with ESCC. Moreover, both plasma D-dimer and serum albumin are routinely measured in clinical practice, and the measurements are convenient, low-cost, and repeatable. Therefore, the DA score may be a simple and instructive indicator for the prognosis of ESCC patient in the future.

## References

[1] Ferlay J, Shin HR, Bray F, Forman D, Mathers C, Parkin DM (2010). Estimates of worldwide burden of cancer in 2008: GLOBOCAN 2008. Int J Cancer.

[2] Lin Y, Totsuka Y, He Y, Kikuchi S, Qiao Y, Ueda J (2013). Epidemiology of esophageal cancer in Japan and China. J Epidemiol..

[3] Pennathur A, Gibson MK, Jobe BA, Luketich JD (2013). Oesophageal carcinoma. Lancet.

[4] Chen WQ, Zheng RS, Zhang SW, Zeng HM, Zou XN (2014). The incidences and mortalities of major cancers in China, 2010. Chin J Cancer..

[5] Tepper J, Krasna MJ, Niedzwiecki D, Hollis D, Reed CE, Goldberg R (2008). Phase III trial of trimodality therapy with cisplatin, fluorouracil, radiotherapy, and surgery compared with surgery alone for esophageal cancer: CALGB 9781. J Clin Oncol.

[6] Akutsu Y, Matsubara H, Shuto K, Uesato M, Mori M, Hoshino I (2009). Clinical and pathologic evaluation of the effectiveness of neoadjuvant chemoradiation therapy in advanced esophageal cancer patients. World J Surg.

[7] Li BZ, Chen ZL, Shi SS, Feng XL, Tan XG, Zhou F (2013). Overexpression of Cdc25C predicts response to radiotherapy and survival in esophageal squamous cell carcinoma patients treated with radiotherapy followed by surgery. Chin J Cancer..

[8] Liu J, Xie X, Zhou C, Peng S, Rao D, Fu J (2012). Which factors are associated with actual 5-year survival of oesophageal squamous cell carcinoma?. Eur J Cardiothorac Surg.

[9] Ahn HS, Lee HJ, Yoo MW, Kim SG, Im JP, Kim SH (2009). Diagnostic accuracy of T and N stages with endoscopy, stomach protocol CT, and endoscopic ultrasonography in early gastric cancer. J Surg Oncol.

[10] Ando M, Ando Y, Hasegawa Y, Shimokata K, Minami H, Wakai K (2001). Prognostic value of performance status assessed by patients themselves, nurses, and oncologists in advanced non-small cell lung cancer. Br J Cancer.

[11] Forrest LM, McMillan DC, McArdle CS, Angerson WJ, Dunlop DJ (2003). Evaluation of cumulative prognostic scores based on the systemic inflammatory response in patients with inoperable non-small-cell lung cancer. Br J Cancer.

[12] Matsuda S, Takeuchi H, Kawakubo H, Fukuda K, Nakamura R, Takahashi T (2015). Cumulative prognostic scores based on plasma fibrinogen and serum albumin levels in esophageal cancer patients treated with transthoracic esophagectomy: comparison with the Glasgow prognostic score. Ann Surg Oncol.

[13] Bick RL (1992). Coagulation abnormalities in malignancy: a review. Semin Thromb Hemost.

[14] Luzzatto G, Schafer AI (1990). The prethrombotic state in cancer. Semin Oncol.

[15] Sun DZ, Liu L, Jiao JP, Wei PK, Jiang LD, Xu L (2010). Syndrome characteristics of traditional Chinese medicine: summary of a clinical survey in 767 patients with gastric cancer. Zhong Xi Yi Jie He Xue Bao..

[16] Ay C, Dunkler D, Pirker R, Thaler J, Quehenberger P, Wagner O (2012). High D-dimer levels are associated with poor prognosis in cancer patients. Haematologica.

[17] Lippi G, Franchini M, Targher G, Favaloro EJ (2008). Help me, Doctor! My D-dimer is raised. Ann Med..

[18] Diao D, Zhu K, Wang Z, Cheng Y, Li K, Pei L (2013). Prognostic value of the D-dimer test in oesophageal cancer during the perioperative period. J Surg Oncol.

[19] Gupta D, Lis CG (2010). Pretreatment serum albumin as a predictor of cancer survival: a systematic review of the epidemiological literature. Nutr J.

[20] Kaya T, Sipahi S, Karacaer C, Nalbant A, Varim C, Cinemre H (2014). Evaluation of nutritional status with different methods in geriatric hemodialysis patients: impact of gender. Int Urol Nephrol.

[21] Caccialanza R, Palladini G, Klersy C, Cereda E, Bonardi C, Quarleri L (2013). Serum prealbumin: an independent marker of short-term energy intake in the presence of multiple-organ disease involvement. Nutrition..

[22] Huang Y, Liu JS, Feng JF (2015). The combination of preoperative serum C-reactive protein and carcinoembryonic antigen is a useful prognostic factor in patients with esophageal squamous cell carcinoma: a combined ROC analysis. Onco Targets Ther..

[23] Liu L, Zhang X, Yan B, Gu Q, Zhang X, Jiao J (2014). Elevated plasma D-dimer levels correlate with long term survival of gastric cancer patients. PLoS One.

[24] Al Murri AM, Bartlett JM, Canney PA, Doughty JC, Wilson C, McMillan DC (2006). Evaluation of an inflammation-based prognostic score (GPS) in patients with metastatic breast cancer. Br J Cancer..

[25] Ishizuka M, Nagata H, Takagi K, Horie T, Kubota K (2007). Inflammation-based prognostic score is a novel predictor of postoperative outcome in patients with colorectal cancer. Ann Surg.

[26] Leung EY, Scott HR, McMillan DC (2012). Clinical utility of the pretreatment Glasgow prognostic score in patients with advanced inoperable non-small cell lung cancer. J Thorac Oncol..

[27] Nozoe T, Iguchi T, Egashira A, Adachi E, Matsukuma A, Ezaki T (2011). Significance of modified Glasgow prognostic score as a useful indicator for prognosis of patients with gastric carcinoma. Am J Surg.

[28] Dupuy E, Hainaud P, Villemain A, Bodevin-Phedre E, Brouland JP, Briand P (2003). Tumoral angiogenesis and tissue factor expression during hepatocellular carcinoma progression in a transgenic mouse model. J Hepatol.

[29] Buller HR, van Doormaal FF, van Sluis GL, Kamphuisen PW (2007). Cancer and thrombosis: from molecular mechanisms to clinical presentations. J Thromb Haemost.

[30] Tanriverdi O (2014). A discussion of serum albumin level in advanced-stage hepatocellular carcinoma: a medical oncologist’s perspective. Med Oncol.

[31] Feng JF, Zhao Q, Chen QX (2014). Prognostic significance of Glasgow prognostic score in patients undergoing esophagectomy for esophageal squamous cell carcinoma. Saudi J Gastroenterol..

[32] Vashist YK, Loos J, Dedow J, Tachezy M, Uzunoglu G, Kutup A (2011). Glasgow Prognostic Score is a predictor of perioperative and long-term outcome in patients with only surgically treated esophageal cancer. Ann Surg Oncol.

